# Genome-Wide Estimates of Coancestry and Inbreeding in a Closed Herd of Ancient Iberian Pigs

**DOI:** 10.1371/journal.pone.0078314

**Published:** 2013-10-31

**Authors:** María Saura, Almudena Fernández, M. Carmen Rodríguez, Miguel A. Toro, Carmen Barragán, Ana I. Fernández, Beatriz Villanueva

**Affiliations:** 1 Departamento de Mejora Genética Animal, Instituto Nacional de Investigación y Tecnología Agraria y Alimentaria (INIA), Madrid, Spain; 2 Departamento de Producción Animal, Escuela Técnica Superior de Ingenieros Agrónomos, Madrid, Spain; Agriculture and Agri-Food Canada, Canada

## Abstract

Maintaining genetic variation and controlling the increase in inbreeding are crucial requirements in animal conservation programs. The most widely accepted strategy for achieving these objectives is to maximize the effective population size by minimizing the global coancestry obtained from a particular pedigree. However, for most natural or captive populations genealogical information is absent. In this situation, microsatellites have been traditionally the markers of choice to characterize genetic variation, and several estimators of genealogical coefficients have been developed using marker data, with unsatisfactory results. The development of high-throughput genotyping techniques states the necessity of reviewing the paradigm that genealogical coancestry is the best parameter for measuring genetic diversity. In this study, the Illumina PorcineSNP60 BeadChip was used to obtain genome-wide estimates of rates of coancestry and inbreeding and effective population size for an ancient strain of Iberian pigs that is now in serious danger of extinction and for which very accurate genealogical information is available (the Guadyerbas strain). Genome-wide estimates were compared with those obtained from microsatellite and from pedigree data. Estimates of coancestry and inbreeding computed from the SNP chip were strongly correlated with genealogical estimates and these correlations were substantially higher than those between microsatellite and genealogical coefficients. Also, molecular coancestry computed from SNP information was a better predictor of genealogical coancestry than coancestry computed from microsatellites. Rates of change in coancestry and inbreeding and effective population size estimated from molecular data were very similar to those estimated from genealogical data. However, estimates of effective population size obtained from changes in coancestry or inbreeding differed. Our results indicate that genome-wide information represents a useful alternative to genealogical information for measuring and maintaining genetic diversity.

## Introduction

Maintaining genetic variation and controlling the increase in inbreeding are crucial requirements in animal conservation programs. It is also well accepted that managing the rate of inbreeding and coancestry, or equivalently, managing the effective population size (*N_e_*), provides a general framework for controlling the loss of variability and can avoid or alleviate the reductions in viability and fertility; i.e., inbreeding depression [1].

In the specific context of livestock species, the maintenance of genetic diversity is critical for addressing current and future challenges to food security, for rural development and for the environment as stressed in the Interlaken Declaration on Animal Genetic Resources (FAO, 2007). Among the porcine breeds, the Iberian breed (*Sus scrofa meridionalis*) is the most emblematic in the Mediterranean area and one of the most important worldwide from an economic point of view [2]. Its meat has a high proportion of intramuscular fat with a rich content of unsaturated fatty acids which leads to highly prized products of extraordinary quality [3]. The breed also has an important role in shaping and maintaining the Dehesa, a richly diverse agrosilvopastoral system of great economic and social importance in the southwest of the Iberian Peninsula [4]. Iberian pigs are well adapted to the strong oscillations in feeding and climate conditions, typical of this geographical area, because of their capacity to accumulate fat.

At the beginning of 1960′s, Iberian pig populations started to suffer a severe decline due to (i) depreciation of animal fats; (ii) introduction of foreign breeds strongly selected for meat production; (iii) outbreak of African swine fever; and (iv) loss of habitat. Although this trend has been now reversed as a result of the expansion of the market to satisfy demands for high quality meat products, some ancestral strains have disappeared and others are endangered. This raises the necessity of strengthening conservation strategies for maintaining this valuable breed.

One of the varieties of Iberian pigs which is now in serious danger of extinction is the Guadyerbas strain. It is one of the most ancient surviving Iberian strains and currently is the only representative of the black hairless genetic type. The strain has been conserved in an experimental herd as a genetically isolated population since 1944 [4]. Accurate genealogical and performance information for reproductive traits has been recorded since the foundation of the herd and this makes it a genetic resource of exceptional value. Due to its particular characteristics, the strain has provided the basic material for numerous genetic studies including analyses of: (i) genetic variability and conservation strategies [4], [5], [6]; (ii) genomic regions associated to different traits [7], [8], [9]; (iii) detection of copy number variants [10]; and (iv) massive sequencing data [2], [11].

Traditionally, rates of coancestry and inbreeding have been measured using pedigree information or a limited number of molecular markers when genealogy was not available. However, the value of classical markers (e.g. microsatellites) has been questioned [12], [13]. Different estimators of genealogical coancestry from molecular markers have been developed but results have been also unsatisfactory [14], [15]. In addition, the assumption that genealogical coancestry is the best parameter for summarizing genetic diversity has also been questioned with the advent of the new genomic technologies [16], [17], [18]. High throughput sequencing and genotyping methods allow us to identify and type thousands of single nucleotide polymorphisms (SNPs) that cover the whole genome and that account for relevant selective variation that microsatellite markers are unable to account for.

In the present study we have evaluated the usefulness of genome-wide data obtained with the Illumina PorcineSNP60 BeadChip for maintaining genetic variability using the conserved population of Guadyerbas pigs. Molecular estimates of coancestry and inbreeding based on SNP data were compared with estimates obtained from pedigree information and from microsatellite data. We have also evaluated the performance of high density SNP data when used in the management of the population for maximizing genetic variability as an alternative to the use of genealogical information.

## Materials and Methods

### Ethical Statement

The current study was carried out under a Project License from the INIA Scientific Ethic Committee. Animal manipulations were performed according to the Spanish Policy for Animal Protection RD1201/05, which meets the European Union Directive 86/609 about the protection of animals used in experimentation. We hereby confirm that the INIA Scientific Ethic Committee, which is the named IACUC for the INIA, specifically approved this study.

### Animal Population

Data from Guadyerbas animals maintained at the CIA ‘El Dehesón del Encinar’ (Oropesa, Toledo, Spain) were available for the study. The herd was established in 1944 and has been maintained in isolation to date under a genetic conservation program [4]. The complete genealogy is available and comprises about 25 generations (67 years). It includes information on 1,178 animals born from 1947 to 2011. A total of 197 sires and 467 dams were used across years. The number of animals that founded the herd were 24 (4 males and 20 females). Animals for which DNA samples were available included 86 males and 141 females born between 1992 and 2011 (about 6 generations).

### SNP Chip Genotypes

The Illumina Porcine SNP60 Bead Chip comprises 62,163 probes which are distributed along 18 autosomal and two sex chromosomes. DNA was extracted from blood samples according to a standard phenol/chloroform protocol, and DNA concentration was quantified using a NanoDrop 1000 spectrophotometer. DNA samples were hybridized with the Porcine SNP60 BeadChip and images were scanned by an external service (Universidad Autónoma de Barcelona, Spain). Genotyping data from the 227 Guadyerbas animals were initially tested for quality using the Illumina Genome Studio Genotyping Module software. In a first step, 241 additional samples from other strains of the Iberian breed were included in the analysis of genotypes. The inclusion of these additional samples allowed us to increase the power of clustering genotypes and detect those SNPs that are segregating in the Iberian breed (it should be noted that this breed was not considered in the design of the chip). Thus, a total of 468 samples were used to check the quality of genotyping data. We used standard parameters for quality control in order to identify SNPs performing incorrectly, i.e. those that failed due to technical reasons or showed a large number of inconsistencies with the genealogy. With the aim of maintaining as much information as possible, quality filters were applied in two sequential steps. We first filtered the SNPs performing incorrectly and reanalysed the data. After that, samples which did not satisfy the quality criteria were also removed. We also excluded SNPs unmapped (5,217) and those mapped on sex chromosomes (1,550 SNPs) according to the last version of the porcine gene annotation (Sscrofa 10.2). Quality control criteria were applied in sequence and included Call Frequency (proportion of samples with genotype at each locus), Gen Train Score (quality of the probe that determines the shape and separation of clusters), AB R Mean (genotype signal intensity), MAF (frequency of the less common allele) and number of inconsistencies with the genealogy. Table S1 gives filtering details and specifies the threshold for each criterion and the number of SNPs removed at each step. Genotypes for SNPs that, satisfying quality control, showed inconsistencies with the genealogy (3,502 genotypes) were assumed to be unknown. Also, eight Guadyerbas samples were eliminated due to inconsistencies with the genealogy (these samples showed more than 1,000 Mendelian inheritance errors, while the maximum number of inconsistencies for the rest of the samples was 6) or to technical problems. Thus, the final number of Guadyerbas samples and autosomal SNPs available for the analysis were 219 and 35,519, respectively.

We also performed the analyses including SNPs showing a large number of inconsistencies (i.e., >9) with the genealogy and the 3,502 genotypes of SNPs with ≤9 inconsistencies. These extra analyses were carried out to mimic situations where genealogy is unavailable. Genotypic data are available on request from the authors.

### Microsatellite Genotypes

Thirty of the Guadyerbas animals genotyped with the SNP chip had also genotypes available for microsatellite markers from a previous study [14]. We used this subsample of the population for comparing molecular estimates obtained with the SNP chip with those obtained with microsatellites. We considered all microsatellites including those monomorphic in Guadyerbas but polymorphic in the Iberian breed. The microsatellite loci analyzed included those used by Toro et al. [14] plus 7 additional loci (i.e., a total of 56 markers were used in the analysis). Microsatellite loci were distributed across the 18 autosomal chromosomes as follows: chromosome 1 (S0113, S0155), chromosome 2 (S0226, SW240, SW395), chromosome 3 (S0002, SW72), chromosome 4 (S0001, S0097, S0214, S0301, SW2404, SW445, SW839, AFABP, S0073, S0217, SW969), chromosome 5 (IGF1, S0005, SW413), chromosome 6 (S0228, SW1057, SW122, SW2419, SW1881), chromosome 7 (S0025, SW1369, SW632), chromosome 8 (S0178, S0225), chromosome 9 (SW1349, SW911, SW749), chromosome 10 (S0038, S0070, SW951), chromosome 11 (S0071, S0835, SW703), chromosome 12 (S0090, S0106, SW874), chromosome 13 (S0219, S0291, S0068), chromosome 14 (SW210, SW857), chromosome 15 (SW1111, SW936), chromosome 16 (S0026, S0061), chromosome 17 (SW1920, SW24), and chromosome 18 (S0120, SW787). Diversity parameters for the microsatellite markers are given in Table S2.

### Inbreeding and Coancestry Coefficients

Following Malécot [19], the molecular coancestry coefficient (*f_M_*) between two individuals is defined as the probability that two alleles taken at random at any locus, one from each individual, are identical in state. Similarly, the molecular inbreeding coefficient (*F_M_*) of an individual is defined as the probability that the two alleles at any locus are identical in state. The molecular coancestry coefficient between individuals *i* and *j* was calculated as 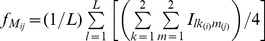 where *L* is the number of markers (SNPs or microsatellites) and *I_lk(i)m(j)_* is the identity of the *k^th^* allele from individual *i* with the *m^th^* allele from the animal *j* at locus *l*, that takes a value of 1 if alleles are identical and 0 if they are not. Unless otherwise stated, estimates given for coancestry included self-coancestries. Inbreeding coefficient for individual *i* was calculated as *F_Mi_* = 2*f_Mii_* –1, where *f_Mii_* is the molecular self-coancestry. Thus, *F_Mi_* was estimated as the proportion of homozygous genotypes. Estimates of average molecular coancestry and inbreeding were also obtained for each autosome separately. The correlation between the molecular and genealogical coefficients was calculated for the 18 autosomes jointly and separately.

Genealogical coancestry (*f_G_*) and inbreeding (*F_G_*) coefficients were calculated using all pedigree information that had been recorded since the foundation of the herd. Estimates of both coefficients were obtained using the ŔTools software (Ricardo Pong-Wong, personal communication) which is based on the algorithm of Meuwissen and Luo [20].

### Rates of Change in Inbreeding and Coancestry and Effective Population Size

Molecular inbreeding and coancestry refer to identity by state (IBS) whereas genealogical inbreeding and coancestry refer to identity by descent (IBD). Thus, molecular and genealogical coefficients are at different scales. However, the rate at which both increase is expected to be the same. For instance, the relationship between molecular and genealogical inbreeding coefficients at time *t* is given by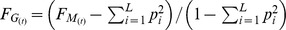 where *p_i_* is the frequency for marker allele *i* in the base population [21]. Note that 
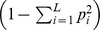
 is the expected heterozygosity in the base population. Substituting this equation into the equation for the rate of genealogical inbreeding (i.e., 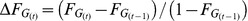), leads to Δ*F_G(t)_ = *Δ*F_M(t)_*, where Δ*F_M_* is the rate of molecular inbreeding. The same applies to rates of change in coancestry. Rates of change in (molecular and genealogical) coancestry per year (Δ*f_M_*_(*y*)_ and Δ*f_G_*_(*y*)_) were computed by regressing the natural logarithm of (1−*f*) for each pair of individuals on year of birth. The slopes of these regressions are approximately equal to −Δ*f_M_*_(*y*)_ and −Δ*f_G_*_(*y*)_ [22]. Inbreeding rates (Δ*F_M(y)_* and Δ*F_G(y)_*) were estimated in the same way, i.e. by regressing the natural logarithm of (1−*F*) on year of birth. Rates of change in coancestry and inbreeding per generation were calculated as *L*Δf*_M_*_(*y*)_, *L*Δ*f_G_*_(*y*)_, *L*Δ*F_M_*_(*y*)_ and *L*Δ*F_G_*_(*y*)_, where *L* is the generation interval which has been estimated in three years. Finally, the effective population size was estimated from the rate of change in coancestry (*N_e_*__*f = *_1/2Δ*f*) and from the rate of change in inbreeding (*N_e_F_*_ = _1/2Δ*F*) per generation using both molecular and genealogical data.

### Mating Criteria

From 1982 the mating strategy followed in the conservation program of Guadyerbas has been to perform minimum coancestry matings, i.e. the average coancestry between the couples is minimized. This is the most appropriate mating strategy for maintaining diversity, at least in the short term [23]. To date, the coancestry minimized has been the genealogical coancestry. However, better results in terms of maintaining genetic variability in the population could be obtained if molecular coancestry calculated from high density markers (i.e. the SNP chip) is minimized instead. With the aim of testing this hypothesis we used empirical data from individuals born in a single generation (a total of 12 males and 47 females born between 1997 and 1999). We used two optimization criteria to perform the matings: minimizing molecular coancestry (*MC_M_* criterion) or minimizing genealogical coancestry (*MC_G_* criterion). In both cases, the restrictions imposed in the optimization included that each female was mated with one male and that 11 out of the 12 males were mated with 4 females and the remaining male was mated with 3 females. This implies a balanced design, ensures that all individuals contribute and mimics what is performed in practice. We used a custom Fortran program to carry out the optimizations that were based on a linear programming algorithm. Average genealogical and molecular coancestries of the couples resulting from the optimization were calculated in order to elucidate which criterion will lead to the lowest (molecular and genealogical) coancestry, and therefore, to the highest genetic variability maintained.

## Results

Although the number of SNPs varied considerably across chromosomes, the density was similar and ranged from 12.6 (chromosome 1) to 17.2 (chromosome 18) SNPs per Mb (Table 1). All chromosomes showed a high proportion of monomorphic SNPs. Note that after removing all monomorphic SNPs for the Iberian breed (Table S1), 16,429 SNPs remained monomorphic for the Guadyerbas strain (i.e. 46%). Average MAF was 0.14±0.02 and average observed heterozygosity was 0.54±0.06 (Table 1).

**Table 1:** Summary statistics of SNPs across chromosomes.

Chrom	*N* SNPs	*N* poly	MAF	*Het*	Density	*f_M_* a	*ρf* b	*F_M_* a	*ρ_F_* b
1	3,953	2,501	0.16	0.63	12.54	0.78	0.4	0.78	0.1
2	2,461	1,188	0.12	0.48	15.18	0.78	0.4	0.77	0.11
3	1,868	1,139	0.16	0.61	12.91	0.78	0.46	0.78	0.28
4	2,337	1,269	0.14	0.54	16.29	0.82	0.57	0.82	0.24
5	1,567	917	0.16	0.59	14.07	0.79	0.54	0.78	0.27
6	2,152	1,107	0.13	0.51	13.64	0.83	0.4	0.82	0.07
7	2,295	1,356	0.14	0.59	17.04	0.81	0.46	0.8	0.1
8	2,064	909	0.11	0.44	13.94	0.86	0.57	0.85	0.48
9	2,308	1,309	0.15	0.57	15.04	0.8	0.46	0.79	0.13
10	1,218	734	0.16	0.60	15.67	0.79	0.61	0.78	0.37
11	1,410	800	0.16	0.57	16.08	0.79	0.5	0.78	0.13
12	1,022	537	0.15	0.53	16.11	0.81	0.55	0.8	0.32
13	2,802	1,258	0.12	0.45	12.82	0.84	0.44	0.83	0.21
14	2,530	1,193	0.11	0.47	16.45	0.86	0.42	0.85	0.11
15	2,099	1,031	0.13	0.49	13.33	0.82	0.54	0.82	0.25
16	1,334	611	0.13	0.46	15.36	0.83	0.47	0.83	0.32
17	1,054	574	0.11	0.54	15.26	0.84	0.37	0.83	0.23
18	1,045	657	0.17	0.63	17.16	0.78	0.45	0.74	0.27
Ave c	1,973	1,061	0.14	0.54	14.94	0.81	0.48	0.80	0.22
SD	753	450	0.02	0.06	1.48	0.03	0.07	0.03	0.11

For each chromosome (Chrom), the number of SNPs with reliable genotypes, number of polymorphic loci (*N* poly), average MAF and heterozygosity (*Het*) are indicated. Density refers to the average number of SNPs per 1 Mb distance. Results were obtained using the 219 Guadyerbas animals and the Sscrofa 10.2 assembly.

a Average coefficient of molecular coancestry (*f_M_*) and inbreeding (*F_M_*).

b Correlation between molecular and genealogical coancestry (*ρ_f_* ) and inbreeding (*ρ_F_*).

c Average metrics for the 18 autosomes.

**Figure 1:**
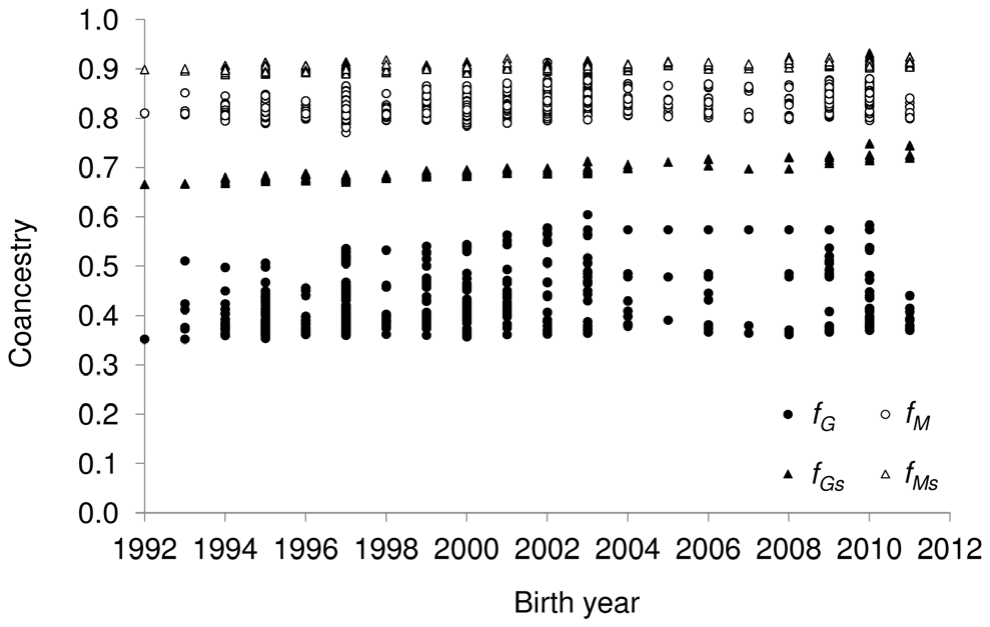
Genealogical (*f_G_*) and molecular (*f_M_*) coancestry coefficients for pairs of individuals born each year. Self-coancestries (*f_Gs_*, *f_Ms_*) are also represented.

Average molecular coancestry and inbreeding coefficients across years (0.82±0.015 and 0.81±0.016, respectively) were almost double that genealogical coefficients (0.42±0.047 and 0.39±0.037) (Figure 1). However, as expected, rates of change in coancestry and in inbreeding and *N_e_* derived from both types of information (i.e. molecular or genealogical) were very similar (Table 2). Coancestry increased at a rate of 3% per generation, whereas inbreeding increased at a rate of 5% and thus, estimates of *N_e_* calculated from changes in coancestry or inbreeding differed (Table 2). When calculated from changes in coancestry, *N_e_* was about 10 animals using either *f_G_* or *f_M_*. When calculated from changes in inbreeding, *N_e_* was about 17 animals. The correlation between SNP-based molecular and genealogical estimates of coancestry (*ρ_f_* ) and inbreeding (*ρ_F_* ) was high, especially for coancestry (0.90 and 0.85 for coancestry, including and excluding self-coancestries, respectively; and 0.68 for inbreeding). These results are very similar to those estimated when SNPs and genotypes showing inconsistencies with the genealogy were included in the analyses. The correlation between both datasets (excluding or not inconsistencies with the genealogy) was 0.97 for coancestry and 1.00 for inbreeding. Results presented henceforth refer to the more stringent filtered dataset (see Table S1).

**Table 2:** Rates of coancestry and inbreeding per year and per generation and corresponding estimates of effective population size.

	Δ*f*_(*y*)_ a	Δ*f* b	*N_e_f_* c	Δ*F*_(*y*)_ a	Δ*F* b	*N_e_F_* c
*Genealogical*	0.0165	0.0495	10.1	0.0100	0.0300	16.7
*SNPs*	0.0160	0.0480	10.4	0.0095	0.0285	17.5

Results were obtained using the 219 Guadyerbas animals.

a Rate of coancestry (Δ*f*_(*y*)_) and inbreeding (Δ*F*_(*y*)_) per year.

b Rate of coancestry (Δ*f*) and inbreeding (Δ*F*) per generation.

c Effective population size calculated from the rate of coancestry (*N_e_f_*) or the rate of inbreeding inbreeding (*N_e_F_*) per generation.

**Figure 2:**
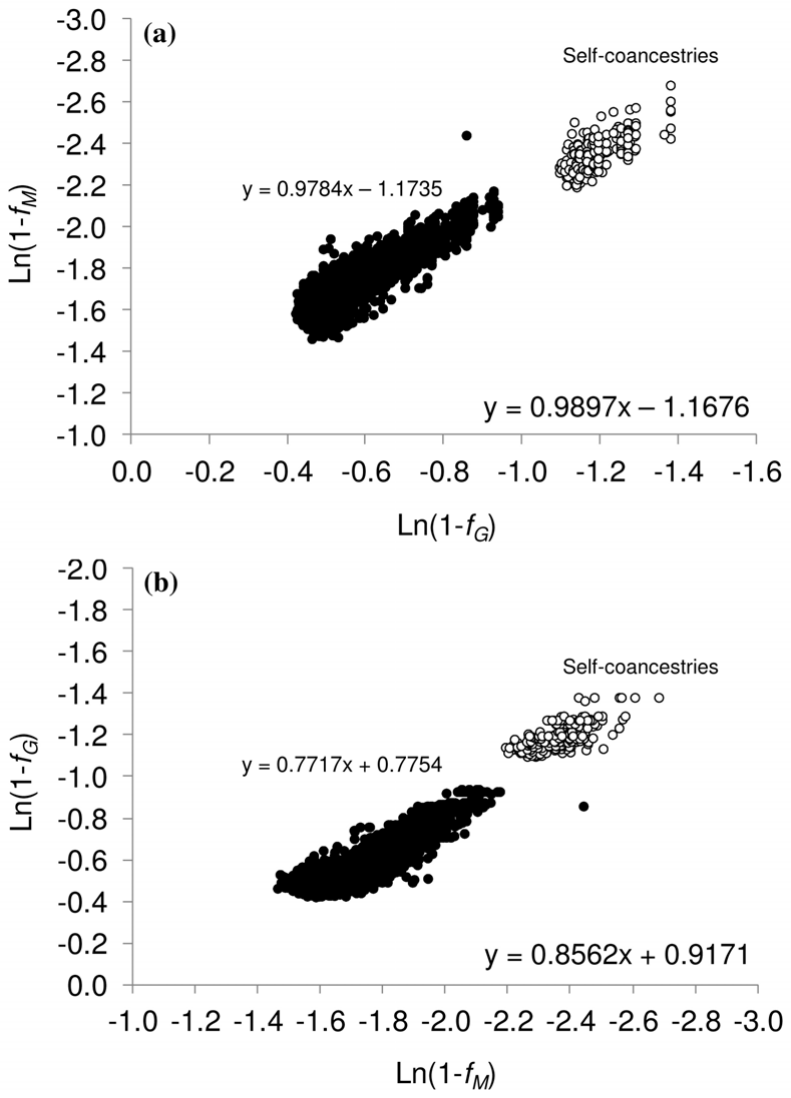
Predictions of SNP-based molecular coancestry (*f_M_*) and genealogical coancestry (*f_G_*) . (A) Predictions of SNP-based molecular coancestry from genealogical coancestry. (B) Predictions of genealogical coancestry from molecular coancestry. The dataset included the 219 Guadyerbas animals with SNP genotypes available. The complete pedigree information was used for the estimation of genealogical coefficients. Regression equations (including and excluding self-coancestries) are indicated.

In order to investigate the accuracy of predicting genealogical coancestry from genome-wide data we performed regressions of *Ln*(1−*f_G_*) on *Ln*(1−*f_M_*). Similarly, the accuracy of predicting molecular coancestry from genealogical data was investigated by performing regressions of *Ln*(1−*f_M_*) on *Ln*(1−*f_G_*). Transforming *f* to *Ln*(1−*f*) permits a meaningful comparison between molecular and genealogical coefficients, given that correcting for base population allele frequencies is not possible. The results show that genealogical coancestry was a very good predictor of molecular coancestry (slope = 0.99 and 0.98 including or not self-coancestries, respectively) as expected, but also, and contrary to what it has been observed in previous studies using microsatellites, molecular coancestry was very accurate in predicting genealogical coancestry (slope = 0.86 and 0.77, including or not self-coancestries, respectively; Figure 2).

The average molecular coancestry and inbreeding coefficients were very similar across chromosomes and ranged from 0.78 to 0.86 and from 0.74 to 0.85, respectively. Nevertheless, the correlation between molecular and genealogical coancestry, and particularly, between molecular and genealogical inbreeding varied widely across chromosomes (Table 1). The latter ranged from less than 0.1 for chromosomes 1 and 6 to about 0.5 for chromosome 8. This wide range observed is not apparently related to the number of SNP, the density of SNP across chromosomes or the number of polymorphic SNP (Table 1).

**Figure 3:**
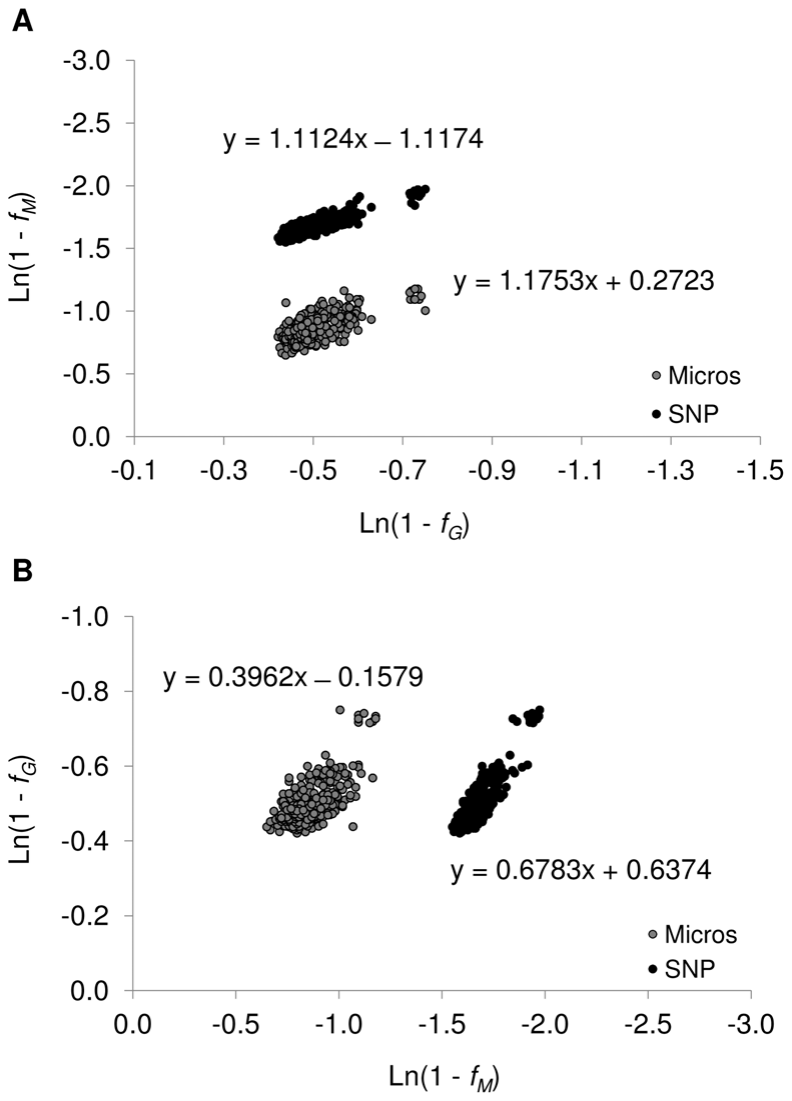
Predictions of SNP-based and microsatellite-based molecular coancestry (*f_M_*) and genealogical coancestry (*f_G_*). (A) Prediction of SNP-based and microsatellite-based molecular coancestry from genealogical coancestry. (B) Prediction of genealogical coancestry from both types of molecular coancestry. The dataset included only the 30 individuals with SNP and microsatellite genotypes available. The complete pedigree information was used for the estimation of genealogical coefficients. Regression equations refer to pairwise coancestries, excluding self-coancestries.

In the group of animals for which microsatellite genotypes were also available, the correlation between genealogical and molecular coancestry (excluding self-coancestries) was substantially higher when the latter was calculated using the SNP chip (0.86) than when calculated using microsatellites (0.67). The correlation between both molecular estimates was as high as 0.86. The prediction of genealogical coancestry from SNPs was more accurate (slope = 0.68, *R^2^* = 0.76) than that from microsatellites (slope = 0.40, *R^2^* = 0.47). As it was observed from the analysis using the whole dataset, the prediction of molecular coancestry from genealogical coancestry was more precise than the prediction of genealogical coancestry from molecular coancestry (Figure 3). Average molecular estimates of coancestry and inbreeding were much lower when using microsatellites than when using SNPs (Table 3). The variance of these estimates were very low (Table 3), ranging from 0.0001 (molecular SNP-based *F*) to 0.006 (genealogical *f*).

**Table 3:** Mean and variance of coancestry and inbreeding coefficients calculated using genealogical, microsatellite or SNP data.

	Genealogy			Microsatellites			SNPs		
	*f_All_* a	*f_Noself_* b	*F* c	*f_All_* a	*f_Noself_* b	*F* c	*f_All_* a	*f_Noself_* b	*F* c
Mean	0.41	0.39	0.35	0.59	0.57	0.55	0.82	0.81	0.80
Variance	0.0061	0.0012	0.0002	0.0040	0.0017	0.0033	0.0006	0.0002	0.0001

Notice that inbreeding coefficients provide equivalent information than self-coancestries. Results were obtained when using the subset of 30 Guadyerbas animals genotyped for both microsatellite and SNP markers.

a Average coancestry coefficient including self-coancestries.

b Average coancestry coefficient excluding self-coancestries.

c Average inbreeding coefficient.

Results derived from the comparison between management criteria for minimizing coancestry indicated that average levels of coancestry between couples were practically identical when minimizing molecular coancestry (*f_M_* = 0.797 and *f _G_* = 0.378) or minimizing genealogical coancestry (*f_ M_* = 0.803 and *f_ G_* = 0.374). Standard deviations of the pairwise coancestries were lower than 0.016 in all four cases. However, the couples chosen using each criterion only coincided in 30% of the cases.

## Discussion

We have compared estimates of coancestry and inbreeding obtained from genome-wide SNP data with those obtained from pedigree information in a closed herd of Iberian pigs and found a high correlation between both types of coefficients. This contrasts with results from previous studies that reported weak correlations between genealogical and molecular coefficients computed from traditional markers [24], [25] and that questioned the efficiency of using molecular data for the maintenance of genetic diversity [12]. We also have shown that, as expected, genealogical coancestry is very good predictor of molecular coancestry but also, and contrary to what it has been observed in previous studies, molecular coancestry computed from thousand of markers is very accurate in predicting genealogical coancestry. Our results indicate thus the necessity of reviewing the paradigm that genealogy is the best parameter for measuring diversity.

Molecular coancestry and inbreeding coefficients were much higher than pedigree-based coefficients. Both types of coefficients were estimated on a different scale, as IBS (molecular estimates) differs from IBD (genealogical estimates). Many estimators have been proposed in the past for correcting molecular coancestry and inbreeding for the homozygosity existing in the base population in order to reflect only IBD [15]. If this were possible, genealogical and molecular coefficients would be expressed in the same scale. However, these methods do not generally work appropriately when as usual there is no information on the allele frequencies in the base population [14]. By transforming the coefficients as described earlier (see Methods), the comparison between molecular and genealogical estimates was meaningful.

Although, as expected [14], molecular and genealogical rates at which both coancestry and inbreeding increase were very similar, Δ*f* and Δ*F* differed (both using molecular or genealogical data). Consequently, estimates of *N_e_* obtained from changes in coancestry or from changes in inbreeding differed. The expectation is that *N_e_f_* and *N_e_F_* are equal under random mating [26] which is not the case in the population analyzed here. The population of Guadyerbas has been managed for generations by performing matings based on minimizing genealogical coancestry and thus mates between relatives have been less frequent than those expected by random, leading to estimates higher for Δ*f* than for Δ*F*. Under this scenario of non-random mating, the loss of genetic variability is better measured with the rate of coancestry than with the rate of inbreeding because inbreeding depends on the specific mating decisions taken [26]. Therefore, the effective population size for the Guadyerbas strain should be considered as low as ten animals. This low population size can explain the high proportion of monomorphic SNPs observed in this strain when compared to other Iberian strains.

The stronger correlation between SNP-based molecular and genealogical coancestries observed in the whole dataset when compared with that for inbreeding may be due to the higher dispersion of genealogical coancestry coefficients (range = 0.41) in comparison to genealogical inbreeding coefficients (range = 0.16). Slate et al. [25] developed theoretical predictions for the correlation between molecular and genealogical inbreeding and compared these predictions with those observed for different vertebrate populations of 12 species using microsatellites. They concluded that *ρ_F_* is highly dependent on the variance of genealogical inbreeding. Other authors have emphasized this dependency rather than focussing on the number of markers and their frequencies for achieving high correlations [24], [27], [28]. In the present study, the variance of genealogical inbreeding was 0.0014, which following Slate et al. [25] would lead to a *ρ_F_* considerably lower than that observed in Guadyerbas (see their Table 1). The same argument would apply to coancestry, for which the variance was also low (0.0022). This suggests that when thousands of markers are used, the variance of genealogical coefficients is not the main factor determining the magnitude of the correlation between molecular and genealogical coefficients. This is supported by the fact that for the subset of 30 Guadyerbas individuals genotyped for both types of markers the correlation between SNP-based and genealogical coancestries was substantially higher than that between microsatellite-based and genealogical coancestries.

Genealogical inbreeding (or coancestry) coefficients are directly related to the average genomic homozygosity and therefore they are expected to be good predictors of molecular coefficients as we have shown here. Previous studies with classical markers such as microsatellites [15], [25], [27] have shown that the opposite is not necessarily true, i.e. that molecular coancestry is not a good predictor of genealogical coancestry. In the present study we have shown that when molecular coefficients are estimated with large numbers of markers they are good predictors of genealogical coefficients. It was also clear that the prediction of genealogical coancestry from SNP-based molecular coancestry was more accurate than that from microsatellite-based coancestry.

The results derived from this work do not only apply to Iberian pigs but also to endangered populations from any species for which dense-SNP information is available.

Pedigree information is difficult and costly to obtain in practice and usually unavailable or unreliable in endangered populations. Genomic approaches have the potential to transform the management of populations in different manners for conservation purposes. The main advantage of SNP chips is that genetic information can be obtained from any animal for which DNA samples are available. The decreasing price of these chips will allow genomic techniques to be affordable and cheaper than recording the pedigree in the near future.

The development of high throughput genotyping techniques has transformed the paradigm that genealogy is the best parameter to measure genetic diversity. Recent studies have emphasized the benefits of these techniques as a powerful tool for analyzing genetic diversity [16], [29], [30]. Having taken into account this new scenario, de Cara et al. [17] performed simulations to evaluate the use of genome-wide SNP markers for maintaining genetic diversity in conservation programs based on optimizing the contributions of parents that minimize global coancestry. Their main conclusion was that management based on molecular coancestry maintains at least equal and usually higher levels of genetic diversity than management based on genealogical coancestry. This is in contrast with results from previous studies using microsatellites that indicated that the exclusive use of molecular markers is of very limited value for maintaining diversity [12]. Although our results showed that management minimizing both molecular and genealogical coancestry gave the same result in terms of average coancestry, the couples chosen by each criterion only coincided in 30% of the cases. This is due to the fact that the molecular coancestry has the ability to discriminate among individuals with the same degree of genealogical coancestry. However, the management strategy employed (i.e. matings of minimum coancestry) led to a reduced variance of coancestry and for this reason the advantage of using molecular coancestry is not translated into practice in this particular case.

An advantage of using genome-wide SNP data is the possibility of determining genetic diversity across chromosomes. Although the average molecular coancestry and inbreeding were similar across autosomes, correlations between molecular and genealogical coefficients varied widely. Similar results were obtained by Silió et al. [31] in a different Iberian strain (Torbiscal) which originated from the mixture of four ancient Iberian pig strains including Guadyerbas. Their lowest correlations between molecular and genealogical inbreeding were found for chromosomes 1, 6 and 11 for which we also found low correlations. The discrepancies across chromosomes may be attributed to linkage disequilibrium arising from selection and/or genetic drift. This strain has been artificially selected for hair colour and coat colour in the past [32]. This could have affected particular genome regions associated with this trait as suggested by Silió et al. [31]. In addition, the bottleneck suffered by the Guadyerbas strain at the begining of this decade may have affected linkage disequilibrium differentially in different chromosomes.

The population analyzed in this study had a complete and accurate pedigree available, a very unusual situation. Here, we have shown that in the absence of complete or reliable pedigree, the usefulness of molecular information from SNP chips to maintain genetic diversity is indisputable because of its straightforward applicability and reasonable cost. In scenarios where reliable pedigree data are available, genome-wide information can provide a more detailed view of the relationships among individuals than genealogical information alone.

## Supporting Information

Table S1 Quality control criteria, thresholds used to filter genotypic data, and number of SNPs discarded.
(DOCX)

Table S2 Summary statistics for the 56 microsatellite loci typed for 30 Guadyerbas individuals.
(DOCX)
