# Supplementary material for: Genome-Wide Estimates of Coancestry and Inbreeding in a Closed Herd of Ancient Iberian Pigs
Source: PLoS One. 2013 Oct 31;8(10):e78314. doi: 10.1371/journal.pone.0078314 (PMC3814548; doi:10.1371/journal.pone.0078314)
Supplement: Table S1 — Quality control criteria, thresholds used to filter genotypic data, and number of SNPs discarded. (DOCX) [file pone.0078314.s001.docx]

Table S1. Quality control criteria, thresholds used to filter genotypic data, and number of SNPs discarded.

| Criterion | Threshold applied | Number of SNPs discarded |
| --- | --- | --- |
| Call frequency | < 0.99 | 2,961 |
| GenTrainScore | < 0.70 | 871 |
| AB R Mean | < 0.35 | 422 |
| Pedigree inconsistencies | > 9 | 14 |
| MAF ^a^ | = 0 | 15,574 |
| Others^b^ |  | 35 |
| Total SNPs excluded |  | 19,877 |

The application of the filters was sequential. Most of pedigree inconsistencies were removed at the three initial steps, except 14 probes that were discarded a posteriori. ^a^Monomorphic SNPs (MAF = 0) for all the Iberian strains analyzed were removed.

^b^SNPs excluded due to other technical problems.
