# Supplementary material for: Genome-Wide Estimates of Coancestry and Inbreeding in a Closed Herd of Ancient Iberian Pigs
Source: PLoS One. 2013 Oct 31;8(10):e78314. doi: 10.1371/journal.pone.0078314 (PMC3814548; doi:10.1371/journal.pone.0078314)
Supplement: Table S2 — Summary statistics for the 56 microsatellite loci typed for 30 Guadyerbas individuals. (DOCX) [file pone.0078314.s002.docx]

Table S2. Summary statistics for the 56 microsatellite loci typed for 30 Guadyerbas individuals.

| Locus | *N_A_*^1^ | Allele Frequency | | | | *H_O_*^2^ | *H_E_*^3^ |
| --- | --- | --- | --- | --- | --- | --- | --- |
| AFABP | 2 | 0.78 | 0.22 |  |  | 0.43 | 0.35 |
| IGF1 | 3 | 0.18 | 0.32 | 0.50 |  | 0.70 | 0.63 |
| S0001 | 2 | 0.67 | 0.33 |  |  | 0.53 | 0.45 |
| S0002 | 2 | 0.53 | 0.47 |  |  | 0.60 | 0.51 |
| S0005 | 4 | 0.13 | 0.18 | 0.35 | 0.33 | 0.87 | 0.73 |
| S0025 | 4 | 0.13 | 0.15 | 0.65 | 0.07 | 0.67 | 0.54 |
| S0026 | 2 | 0.42 | 0.58 |  |  | 0.50 | 0.49 |
| S0038 | 2 | 0.52 | 0.48 |  |  | 0.37 | 0.51 |
| S0061 | 2 | 0.50 | 0.50 |  |  | 0.53 | 0.51 |
| S0068 | 3 | 0.42 | 0.28 | 0.30 |  | 0.70 | 0.67 |
| S0070 | 4 | 0.27 | 0.33 | 0.27 | 0.13 | 0.73 | 0.74 |
| S0071 | 2 | 0.37 | 0.63 |  |  | 0.53 | 0.47 |
| S0073 | 3 | 0.02 | 0.38 | 0.60 |  | 0.47 | 0.50 |
| S0090 | 2 | 0.58 | 0.42 |  |  | 0.50 | 0.49 |
| S0097 | 4 | 0.60 | 0.28 | 0.02 | 0.10 | 0.63 | 0.56 |
| S0106 | 3 | 0.33 | 0.32 | 0.35 |  | 0.77 | 0.68 |
| S0113 | 2 | 0.57 | 0.43 |  |  | 0.60 | 0.50 |
| S0120 | 3 | 0.72 | 0.18 | 0.10 |  | 0.47 | 0.45 |
| S0155 | 3 | 0.20 | 0.63 | 0.17 |  | 0.60 | 0.54 |
| S0178 | 2 | 0.27 | 0.73 |  |  | 0.47 | 0.40 |
| S0214 | 2 | 0.75 | 0.25 |  |  | 0.43 | 0.38 |
| S0217 | 1 | 1.00 |  |  |  | 0.00 | 0.00 |
| S0219 | 1 | 1.00 |  |  |  | 0.00 | 0.00 |
| S0225 | 2 | 0.65 | 0.35 |  |  | 0.57 | 0.46 |
| S0226 | 2 | 0.17 | 0.83 |  |  | 0.27 | 0.28 |
| S0228 | 2 | 0.78 | 0.22 |  |  | 0.37 | 0.35 |
| S0291 | 3 | 0.35 | 0.05 | 0.60 |  | 0.40 | 0.52 |
| S0301 | 1 | 1.00 |  |  |  | 0.00 | 0.00 |
| S0385 | 1 | 1.00 |  |  |  | 0.00 | 0.00 |
| SW1057 | 2 | 0.63 | 0.37 |  |  | 0.60 | 0.47 |
| SW1111 | 3 | 0.22 | 0.72 | 0.07 |  | 0.40 | 0.44 |
| SW122 | 3 | 0.50 | 0.12 | 0.38 |  | 0.57 | 0.60 |
| SW1349 | 3 | 0.07 | 0.92 | 0.02 |  | 0.17 | 0.16 |
| SW1369 | 3 | 0.53 | 0.33 | 0.13 |  | 0.70 | 0.60 |
| SW1881 | 2 | 0.85 | 0.15 |  |  | 0.30 | 0.26 |
| SW1920 | 2 | 0.82 | 0.18 |  |  | 0.30 | 0.30 |
| SW210 | 2 | 0.98 | 0.02 |  |  | 0.03 | 0.03 |
| SW24 | 2 | 0.27 | 0.73 |  |  | 0.33 | 0.40 |
| SW240 | 3 | 0.18 | 0.58 | 0.23 |  | 0.60 | 0.58 |
| SW2404 | 2 | 0.73 | 0.27 |  |  | 0.47 | 0.40 |
| SW2419 | 4 | 0.27 | 0.23 | 0.03 | 0.47 | 0.67 | 0.67 |
| SW395 | 3 | 0.30 | 0.17 | 0.53 |  | 0.70 | 0.61 |
| SW413 | 2 | 0.60 | 0.40 |  |  | 0.53 | 0.49 |
| SW445 | 2 | 0.40 | 0.60 |  |  | 0.40 | 0.49 |
| SW632 | 1 | 1.00 |  |  |  | 0.00 | 0.00 |
| SW703 | 3 | 0.13 | 0.33 | 0.53 |  | 0.53 | 0.60 |
| SW72 | 3 | 0.10 | 0.62 | 0.28 |  | 0.57 | 0.54 |
| SW749 | 2 | 0.32 | 0.68 |  |  | 0.37 | 0.44 |
| SW787 | 3 | 0.62 | 0.03 | 0.35 |  | 0.53 | 0.50 |
| SW839 | 1 | 1.00 |  |  |  | 0.00 | 0.00 |
| SW857 | 2 | 0.20 | 0.18 | 0.62 |  | 0.63 | 0.56 |
| SW874 | 4 | 0.32 | 0.45 | 0.22 | 0.02 | 0.77 | 0.66 |
| SW911 | 2 | 0.90 | 0.10 |  |  | 0.20 | 0.18 |
| SW936 | 4 | 0.23 | 0.18 | 0.37 | 0.22 | 0.83 | 0.74 |
| SW951 | 2 | 0.52 | 0.48 |  |  | 0.43 | 0.51 |
| SW969 | 2 | 0.95 | 0.05 |  |  | 0.10 | 0.10 |

Notice that 6 out of 56 microsatellites were monomorphic.

^1^ Number of alleles.

^2^ Observed heterozygosity.

^3^ Expected heterozygosity.
